# Assessment of the molecular epidemiology and genetic multiplicity of *Listeria monocytogenes* recovered from ready-to-eat foods following the South African listeriosis outbreak

**DOI:** 10.1038/s41598-022-20175-x

**Published:** 2022-11-22

**Authors:** Adeoye John Kayode, Anthony Ifeanyi Okoh

**Affiliations:** 1 Applied and Environmental Microbiology Research Group (AEMREG), Department of Biochemistry and Microbiology, University of Fort Hare, Private Bag X1314, Alice, 5700 South Africa; 2 SAMRC Microbial Water Quality Monitoring Center, University of Fort Hare, Private Bag X1314, Alice, 5700 South Africa; 3 Department of Environmental Health Sciences, College of Medical and Health Sciences, University of Sharjah, Sharjah, United Arab Emirates

**Keywords:** Applied microbiology, Biofilms, Microbial communities, Microbiology

## Abstract

Following the recent listeriosis outbreak in South Africa, this study was carried out to assess the safety level of various common ready-to-eat foods (RTE) obtained from supermarkets and grocery stores in major towns and cities within the Amathole, Chris Hani and Sarah Baartman Districts Municipalities, Eastern Cape Province, South Africa. A sum of 239 food samples was collected from these locations, and *Listeria monocytogenes* (Lm) was isolated in line with the recommended techniques by the International Organization for Standardization EN ISO 11290:2017 parts 1 and 2. Identification of the pathogen and detection of various associated virulence genes was done using Polymerase Chain Reaction (PCR) techniques. From the RTE food samples processed, Lm was detected in 107 (44.77%) of the samples. Russian sausage was the most contaminated (78.57%), followed by sliced polony (61.90%), muffins (58.33%), polony (52.63%), and pies (52.38%), while all vetkoek samples examined were negative for Lm. Although the prevalence of Lm in the food samples was very high, concentrations were generally < 100 CFU/g. Strains of Lm recovered from the RTE foods were predominantly epidemiological strains belonging to serotypes 1/2a, 1/2b and 4b. The prevalence of 10 virulence genes including the *inl*A*, Inl*C, *inl*J, *plc*A, *hly*A*,* *plc*B, *prf*A, *mpl, inl*B*,* and *act*A were detected among Lm isolates. Most of the isolates (69.07%) demonstrated the potential for biofilm formation and were categorized as weak (14.95%), moderate (13.40%) and strong (40.72) biofilm formers. Furthermore, molecular typing revealed high levels of genetic diversity among Lm isolates. The findings of this investigation suggested that the presence of Lm in the RTE foods may constitute potential threats to the food sector and could pose public health hazards to consumers, particularly the high-risk group of the population. We, therefore, recommend that adequate food monitoring for safety and proper regulation enforcement in the food sector must be ensured to avoid any future listeriosis outbreak that could be linked to RTE foods in South Africa.

## Introduction

Food is a substance accommodating vast arrays of nutrients that support microbial proliferation, including the pathogenic species^1^. Over the last decades, several food types have been implicated in listeriosis outbreaks, leading to severe health consequences in various parts of the world^2–6^. This could be attributed to the increasing demand for ready-to-eat (RTE) foods by many individuals, probably because minimal or no preparation is required before consumption as well as changing lifestyles^7^.

*Listeria monocytogenes* (Lm) which causes listeriosis, is one of the leading pathogens that have been implicated in several foodborne outbreaks with an increasing trend of listeriosis since 2008^8–11^. *Listeria* is a Gram-positive, catalase-positive, non-spore-forming bacterium of the family Listeriaceae^12^. The genus currently comprises 28 known species^13–18^*.* Among these species, *L. monocytogenes* infects both humans and animals (most especially ruminants), while *L. ivanovii* is majorly an animal pathogen^15^*.* The pathogen gains access to the humans when contaminated foods containing the pathogen are consumed. The elderly, young and immunocompromised individuals are mostly the high risks group for the pathogen^12,19^. Listeriosis infection manifests symptoms including gastroenteritis, encephalitis, meningitis, septicemia, stillbirths, and abortion in invasive cases. Non‐invasive listeriosis manifests less severe symptoms like fever, diarrhoea, headache, and muscle pain (myalgia) which may ensue after consumption of foods contaminated by large numbers of Lm. In most invasive cases, about 20–30% of deaths are usually recorded^9,12^.

Foodborne listeriosis is commonly associated with RTE foods preserved under refrigeration and foods requiring minimal or no preparation before consumption. Microbiologically, the safety of food is of major significance to the producer as well as to the consumers^7^. However, several studies have reported the isolation of Lm from heat-processed, minimally processed and raw RTE foods including meat and meat products, fish and fish products, sausages, cooked ham, and deli ham^7,20^. Given this, various regulating bodies had set the benchmarks for the acceptable level of Lm in RTE foods based on their potential to support the growth of Lm. States in the EU adopted 100 CFU/g of Lm as the microbiological limit in foods that would not promote the proliferation of the pathogen during storage shelf-life. Zero-tolerance was adopted in 25 g after production, in foods that could promote Lm growth if there are no assurances that the growth would not exceed the maximum of 100 CFU/g during storage. However, the US has implemented a zero-tolerance policy for Lm in foods due to the risks associated with listeriosis^7,12^.

Several approaches, including phenotypic and genotypic determination, have been deployed in epidemiological surveillance and source-tracking listeriosis agents. Lm was classified into 14 serotypes including the recently described serotype 4 h (a novel hypervirulent hybrid belonging to the sub-lineage II)^21^ based on the flagella (H) and somatic (O) antigens^22^. Furthermore, Lm was categorized into five distinct phylogenetic lineages, designated as serogroups I.1 (1/2a-3a), I.2 (1/2c-3c), II.1 (4b-4d-4e), II.2 (1/2b-3b-7), and III (4a-4c)^22,23^. The expression of virulence genes during the cellular infection cycle could be serotype dependent, with serotypes 1/2a, 1/2b, 1/2c and serotype 4b strains frequently associated with food and account for about 95% of the strains isolated from cases of human listeriosis^22,23^.

Some classes of virulence factors referred to as the *Listeria* pathogenicity islands (LIPI) were identified as being responsible for *Listeria* pathogenicity. The LIPI-1 (*act*A, *hly, iap, plc*B, *plc*C*, prf*A and *mpl*), LIPI-2 and the internalin genes (A, B, C and J) are regarded as the major classic LIPI in *Listeria*^22,23^. LIPI-2, a 22 kb gene cluster *is* is composed of several internalin genes^24^. Internalin genes are known to mediate the ability of Lm to invade and cross cellular barriers during infection^25^. Furthermore, the LIPI-3 contains the *IIs*X gene (encoding hemolytic, listeriolysin, and cytotoxic factor) and was found in cases of human listeriosis^23,26^. Also, the LIPI-4 harbouring a six-gene cluster [cellobiose-family phosphotransferase system (PTS)] is usually responsible for cases of neuro-invasive and maternal neonatal infection in humans^27^.

Usually, most listeriosis outbreaks reported have been accompanied by serious health implications and economic losses. The most recent listeriosis outbreak traced to “Polony” in South Africa was accompanied by severe health implications (which led to about 1060 cases and 216 deaths nationwide where 53 cases and 13 deaths were reported in the Eastern Cape Province). Also, the economic losses due to the outbreak valued at about 15 million USD and this necessitated the need for regular surveillance and monitoring to ensure food safety for human consumption^12,28^. Therefore, we carried out a comprehensive assessment of the occurrence, level of contamination, phenotypic and genotypic virulence determination, and the genetic relatedness of the RTE food isolates surveyed in the Eastern Cape Province, South Africa.

## Materials and methods

### Study area and sample collection

The present investigation was carried out within the Amathole, Chris Hani and Sarah Baartman District Municipalities, Eastern Cape Province, South Africa occupying the geographical coordinates “32.5842° S, 27.3616° E, 31.8743° S, 26.7968° E and 33.57° S, 25.36° E on the map respectively. Sampling of RTE foods widely consumed by many individuals in the Republic of South Africa (RSA) was done for eight months (February–September 2019). A sum of 239 samples of various RTE foods from grocery stores/supermarkets was collected at different locations and towns within the selected municipalities. The foods include polony (a soft-textured sausage typically made of pork and beef encased in a vividly hued orange or red skin), fruit salad, potato chips, fried fish, Russian sausage (also known as Kolbasa, made from ground meat—usually beef, poultry or pork, along with salt, spices and other flavourings wrapped in a special casing), red Vienna sausage (meaty, soft, fine textured, red-skinned sausage produced from pork, mechanically deboned chicken, salt, spices, vegetable protein and other ingredients), bread, fried chicken, vetkoek (also known as “Amagwinya/fat cake” a traditional South African fried dough that is crispy outside and fluffy inside often stuffed with sweet or savoury fillings), Meat pies, cupcakes, muffin, assorted sandwiches (consists of two floors made with slices of bread to which is added bacon, cheese, chicken, lettuce and tomato), fruit salad (made from a combination of fruits in a sweet sauce made from juice and honey), Assorted sandwiches (made from cream cheese, lemon rind, bread slices, tomato, lettuce, and sausages) *from grocery* stores/supermarkets were collected at the mid-shelf-life. Foods like potato chips, fried fish, vetkoek and fried chicken were processed by frying. Bread, cupcakes, muffin and meat pies were prepared by baking. Russian sausage and red Vienna sausage were processed either by grilling/roasting/smoking, polony was prepared by the application of medium hot smoke for 2 h and boiling for 90 min at 80 °C. The foods were kept refrigerated in a cool dry place. Each of the samples was aseptically collected from the sampling location and immediately transferred into labelled sterile plastic bags to prevent cross-contamination. The samples were conveyed to the laboratory in iced insulated boxes for analysis within 6 h.

### Quantitative analysis

#### Enumeration of presumptive *Listeria* levels in food samples

The microbial counts of the food samples were enumerated as documented by the International Organization for Standardization EN ISO 11290–2:2017^29^. Twenty-five grams (25 g) of RTE food samples were weighed and stomached (Interscience international BagMixer, France) in 225 ml of BPW (buffered peptone water CM1049 Oxoid Ltd, UK) and were serially diluted in three replicates of ten-fold dilutions. About 0.5 ml of the appropriate dilutions were surface plated on Chromogenic *Listeria* Agar (ISO) Base (CM1084 Oxoid Ltd, UK) supplemented with OCLA (ISO) differential supplement (SR0244E Oxoid Ltd, UK) and selective supplements (SR0226E Oxoid Ltd, UK) and Brilliance *Listeria* Agar Base (CM1080 Oxoid Ltd, UK) supplemented with Brilliance differential supplement (SR0228E Oxoid Ltd, UK) and selective supplements (SR0227E Oxoid Ltd, UK). The plates were incubated at 37 °C between 24 and 48 h in aerobic conditions. Typical colonies (blue or blue-green) obtained were counted and expressed as colony-forming units per gram (CFU/g)^29^.

#### Detection of presumptive *Listeria* isolates

Isolation of Lm was done according to EN ISO 11290-1:2017 guidelines^29,30^. Twenty-five grams of each sample was briefly stomached and pre-enriched in 225 ml of Half-Fraser Broth Base (CM0895) supplemented with half Frazer selective supplement (SR0166E Oxoid Ltd, UK) for primary enrichment (incubation at 30 °C for 24 h) to resuscitate the pathogens. After this, 0.1 ml of pre-enriched samples were added to a 10 ml Fraser Broth Base (supplemented with Frazer selective supplement (SR0156E Oxoid Ltd, UK) for secondary enrichment (24 h at 37 °C). After secondary enrichments, the broths were surface-plated on the media described in section “Enumeration of presumptive *Listeria* levels in food samples”. The plates were incubated aerobically at 37 °C for 24–48 h. About three to five representative distinct colonies (blue, blue-green with or without halos) were subcultured on nutrient agar for purification of the isolates. Pure cultures of presumptive isolates were preserved at − 80 °C in Tryptic Soy Broth with 25% glycerol.

### Qualitative analysis

#### Extraction of genomic DNA

The extraction of genomic DNA was done using a direct boiling method described in our previous study^31^. The DNA template was quantified to determine the concentration in a fluorometer (Invitrogen Qubit fluorometer, Turner BioSystems) and was preserved in Eppendorf tubes at − 20 °C for further analysis.

#### Molecular characterization of *L. monocytogenes*

A 370 base pair of the *prs* gene for *Listeria* genus was amplified by polymerase chain reaction (PCR) in a thermal cycler (BIO-RAD T100) using the primer sets described in Table S1. The cycling condition for the assay was 94 °C—5 min, 33 cycles (94 °C—45 s, annealing 56 °C—30 s, 72 °C—1 min, the final temp of 72 °C—5 min) held at 4 °C. The isolates were further verified to determine if they are Lm using specific primer sets (Table S1) previously described^32^ targeting the invasion-associated protein (*iap*) gene at 131 bp^33^. The cycling condition for the assay was 94 °C—5 min, 35 cycles (94 °C—35 s, annealing 52 °C—30 s, 72 °C—1 min, the final temperature of 72 °C—10 min) held at 4 °C. Referenced strains of Lm (ATCC 19118 and ATCC 7644) were used as a positive control. The PCR product of the targeted genes for Lm was further verified by sequencing. The sequencing was performed bidirectionally, and blast studies were conducted. All electropherograms obtained were edited in Geneious Prime (version 2.6.6) and compared with reference sequences in the National Center for Biotechnology Information GeneBank.

#### Serotyping *L. monocytogenes* isolates

Multiplex PCR technique was adopted for the molecular classification of Lm to various serotypes using the five primer sets previously described^22^. The five primer sets used for this assay were mixed at final concentrations of *prs* 0.2 M; 1 M for *ORF*2819, *ORF*2110, *Imo*0737, and 1.5 M for Imo1118. Serotype 4b was amplified by both the *ORF*2110 and the *ORF*2819 DNA fragment, while serotypes 1/2a and 1/2b were only amplified by the *Imo*0737 and *ORF*2819 DNA fragment respectively. The reaction mix was prepared and amplified using the cycling conditions thus: 94 °C—3 min, 35 cycles (94 °C—24 s, annealing 53 °C—75 s, 72 °C—1.15 min, the final temperature of 72 °C—7 min) held at 4 °C.

#### Detection of virulence markers of *L. monocytogenes* isolates

Ten virulence genes including the *act*A, *hly*A, *plc*A, *prf*A, *plc*B, and *mpl, inl*A, *inl*B, *inl*C, *inl*J of Lm isolates were amplified using the primer sets documented previously^34,35^ as presented in Table SI. Amplification of the gene fragments was assayed using the program described in our previous study^36^.

### Assessment of biofilm-forming potential by the crystal violet (CV) techniques

The microtiter plate biofilm production was employed to evaluate the biofilm-forming ability of Lm isolates. The bacterial cell adherence potentials to the abiotic surface were assessed using freshly grown overnight Lm culture grown in TSB at 37 °C as described in our previous study^31^. The non-inoculated TSB served as negative control while Lm strains (ATCC 7644 Mast diagnostics group Ltd, Merseyside U.K. and ATCC 19118 MediMark ® Europe) served as the positive control. The absorbance was read at 595 nm (Abs@595) by a microtitre photometer (Synergy^TM^Mx Monochromator-Based Multi-Mode Reader w/Time-resolved fluorescence, Biotek Instruments, USA). Abs@595 nm recorded for all positive and negative controls (AbsNC) were computed to obtain the mean and standard deviation. The results obtained were used to categorized Lm isolates as either weak = (AbsNC < Abs@595 nm ≤ 2 × AbsNC), moderate = (2 × AbsNC < Abs@595 nm ≤ 4 × AbsNC) or strong = (4 × AbsNC < Abs@595 nm) biofilm formers^37^.

### Genetic typing of *L. monocytogenes* by Enterobacterial repetitive intergenic consensus (ERIC) PCR

The molecular diversity of the previously identified Lm isolates was determined by ERIC-PCR using the primer sets ERIC-1 5′-ATGTAAGCTCCTGGGGATTCAC-3′ and ERIC-2 5′-AAGTAAGTGACTGGGGTGAGCG-3′^31^. The PCR reaction was prepared in a final volume (25 µL) containing 1 µl of ERIC primers, 12.5 µl master mix (One taq Quick Load 2 × Master mix, New England BioLabs Inc.), 3 µl aliquots of DNA, 0.5 µl of MgCl_2_, buffer, and 6.5 µl of sterile nuclease-free water. The thermal cycling program for the ERIC-PCR was 95 °C—5 min, 35 cycles (94 °C—1 min, annealing 35 °C—1 min, 72 °C—2 min), a final step of 72 °C—10 min and held refrigerated at 4 °C. The reaction products (5–10 µl) were separated in a 1.5% agarose gel stained with ethidium bromide (3 µl) and suspended in a 5 × TBE buffer subjected to 90 V for 240 min using a Bio-Rad electrophoresis machine (Bio-Rad**®** PowerPac™ Basic Power Supply, Singapore). An appropriate volume of DNA ladder ranging from 100 bp and 10 kb was put in the gel. The gel image was captured and recorded using Alliance 4.7 UV trans-illuminator (Alliance XD-79.WL/26MX, France).

#### Digitization and evaluation of intra-species/strain diversity of *L. monocytogenes* isolates

The digitization of all fingerprint images was done by a computer-assisted software analysis (GelJ version 2.0). Determination of the molecular weight and occurrence matrices of the ERIC-PCR bands was done by the UPGMA algorithm (unweighted pair group arithmetic mean) at 1.0% tolerance level for control of quality. The diversity of the isolates was further studied by the presence/absence as the basis for strain homo/heterogeneity (associations) assessment. The dendrogram of two matrices was generated by neighbour-joining (NJ) through a Euclidean similarity index and the relative abundance of strains forming a clade was determined using Paleontological Statistical software version 4.03 (PAST 4.03).

### Statistical analysis

Data obtained were analysed statistically to compare Lm counts in RTE food matrices using one-way analysis of variance (ANOVA). The statistical significance of mean ± SD was considered at (p ≤ 0.05). Spearman’s correlation test was employed to determine the prevalence and association of biofilm-forming Lm isolates recovered with RTE food (Eq. 1). Spearman’s chi-square test (Eq. 2) was used to measure the prevalence and association between categorical variables (food types, serotypes, and genetic virulence determinants). The significant difference was identified at (p ≤ 0.01) and (p ≤ 0.05) as appropriate.

1 $$\rho = 1 - \frac{{6\sum {d_{i}^{2} } }}{{n\left( {n^{2} - 1} \right)}}$$

where *ρ* = spearman’s rank correlation coefficient, n = number of observations, and *d*_i_ = difference between the two ranks of each observation.

2 $$X^{2} = \sum {\frac{{(o - e)^{2} }}{e}}$$

where *X*^2^ = chi square, *O* = observed and *E* = expected.

## Results

### Prevalence and distribution of *L. monocytogenes* in RTE foods

Two hundred and thirty-nine (239) RTE food samples collected from the grocery/retail stores and roadside vendor at different geographical locations in the study area were processed to assess *Listeria* density in the samples (Table 1). The presumptive Lm counts from the RTE food samples analyzed ranged from 1.0 × 10^3^ to 2.7 × 10^6^ CFU/g. The mean ± SD of Lm counts were compared using a one-way analysis of variance (ANOVA) to determine the statistical significance. The analysis revealed that food types influenced the log counts of *Listeria* in RTE foods analyzed. Food types, especially sliced polony, potato chips, fried fish, bread, meat pie, Russian sausages, and cupcake presents higher contamination levels. This suggests that these foods may be at higher risk of Lm contamination among the RTE food samples analyzed. The contamination levels of RTE food samples were categorized into four regimes thus: 0, < 10, 10–100, and > 100 CFU/g. About 73 (30.5%) of the food samples had counts < 10 CFU/g, 25 (10.46%) had counts between 10 and 100 CFU/g while 9 (3.77%) food samples had counts > 100 CFU/g among the RTE food samples analyzed (Table 1).

**Table 1:** Presumptive aerobic plate counts of *Listeria* and contamination levels of RTE food samples analyzed.

Sample types	Number of samples		Counts of *Listeria* in RTE food samples (CFU/25 g)			
	Samples tested	Positive samples for presumptive *Listeria* counts (%)	0	< 10	10–100	> 100
Polony	19	10 (52.63)	9	9	1	0
Sliced polony	21	13 (61.90)	8	5	5	3
Fruit salad	20	10 (50)	10	7	3	0
Potato chips	21	10 (47.62)	11	9	1	0
Fried fish (Snoek)	21	8 (38.10)	13	3	3	2
Russian sausage	14	11 (78.57)	3	6	3	2
Vienna sausage	8	4 (50)	4	3	1	0
Bread	21	6 (28.57)	15	3	3	0
Fried chicken	5	2 (40)	3	1	1	0
Vetkoek	21	0	21	0	0	0
Meat pie	21	11 (52.38)	10	9	2	0
Cupcakes	21	9 (42.86)	12	7	1	1
Muffins	12	7 (58.33)	5	6	0	1
Assorted sandwiches	14	6 (42.86)	8	5	1	0
Total (%)	239	107 (44.77)	132 (55.23)	73 (30.54)	25 (10.46)	9 (3.77)

Contamination is higher (p < 0.01) in specific RTE food (sliced polony, potato chips, fried fish, bread, meat pie, Russian sausages, and cupcake).

Lm was detected in 107 (44.77%) of the RTE food samples (Table 2). The identity of the isolates recovered from the food samples was established by PCR assay. Figure S1a and S1b are images of the PCR gel electrophoresis of the confirmed Lm recovered from RTE food samples. Lm isolates were distributed at various percentiles across the RTE food samples except for vetkoek from which Lm was not detected. Among the food types analyzed, 78.57% of Russian sausage (highest % occurrence) was positive for the presence of Lm, followed by sliced polony (61.90%), muffins (58.33%) and polony (52.63%). Bread had the lowest occurrence (28.57%) followed by fried fish (38.10%), cupcakes and assorted sandwiches had 42.86%. Tables 1 and 2 summarily describe the samples, the prevalence of Lm and the safety levels of RTE food samples analyzed.

**Table 2:** Occurrence, serotypes, phenotypic and genotypic determinants of *L. monocytogenes* virulence recovered from RTE foods.

Sample type	No of RTE foods positive for Lm (%)	No of Lm in RTE food	Distribution of Lm serotypes in RTE foods samples (%)				Genetic determinants of virulence (%)			Biofilm formation (%)			
			1/2a*	1/2b*	4b*	NT*	LIPI-1*	*inl*(A, B, C, J)*	LIPI-1 + *inl* (A, B, C, J)*	Negative	Weak	Moderate	Strong
Polony	10 (52.63)	20	5 (17.24)	12 (10.08)	3 (8.82)	0	14 (11.86)	13 (12.62)	11 (12.64)	4 (6.67)	3 (10.35)	4 (15.38)	9 (11.39)
Sliced polony	13 (61.90)	23	4 (13.80)	14 (11.76)	4 (11.77)	1 (8.33)	15 (12.71)	14 (13.59)	11 (12.64)	8 (13.33)	8 (27.59)	2 (7.69)	5 (6.33)
Fruit salad	10 (50)	30	1 (3.44)	20 (16.81)	7 (20.59)	2 (16.67)	24 (20.34)	29 (28.16)	24 (27.59)	15 (25)	4 (13.79)	3 (11.54)	8 (10.13)
Chips	10 (47.62)	16	3 (10.35)	11 (9.24)	2 (5.88)	–	13 (11.02)	12 (11.65)	11 (12.64)	1 (1.67)	1 (3.45)	2 (7.69)	12 (15.19)
Fried fish (Snoek)	8 (38.10)	21	4 (13.80)	13 (10.92)	4 (11.76)	–	12 (10.17)	9 (8.74)	9 (10.34)	6 (10)	1 (3.45)	1 (3.85)	13 (16.46)
Russian sausage	11 (78.57)	14	3 (10.35)	8 (6.72)	2 (5.88)	1 (8.33)	5 (4.24)	11 (10.68)	5 (5.75)	2 (3.33)	4 (13.79)	3 (11.54)	5 (6.33)
Vienna sausages	4 (50)	4	–	2 (1.68)	2 (5.88)	–	2 (1.69)	3 (2.91)	2 (2.30)	1 (1.67)	1 (3.45)	1 (3.85)^-^	1 (1.27)
Bread	6 (28.57)	11	–	7 (5.88)	3 (8.82)	1 (8.33)	7 (5.93)	11 (10.68)	8 (9.20)	6 (10)	2 (6.90)	2 (7.69)	1 (1.27)
Fried chicken	2 (40)	2	–	2 (1.68)	–	0	1 (0.85)	1 (0.97)	1 (1.15)	0	1 (3.45)	1 (3.85)	0
Vetkoek	0	0	–	–	–	0	0	0	0	0	0	0	0
Meat pie	11 (52.38)	22	6 (20.69)	9 (7.56)	2 (5.88)	5 (41.67)	6 (5.08)	13 (12.62)	8 (9.20)	10 (16.67)	3 (10.34)	4 (15.38)	5 (6.33)
Cupcakes	9 (42.86)	10	1 (3.44)	6 (5.04)	2 (5.88)	1 (8.33)	4 (3.39)	7 (6.80)	4 (4.60)	2 (3.33)	0	3 (11.54)	5 (6.33)
Muffins	7 (58.33)	12	1 (3.44)	8 (6.72)	2 (5.88)	1 (8.33)	7 (5.93)	11 (10.68)	6 (6.90)	4 (6.67)	1 (3.45)	0	7 (8.86)
Assorted sandwiches	6 (42.86)	9	1 (3.44)	7 (5.88)	1 (2.94)	0	5 (4.24)	8 (7.77)	4 (4.60)	1 (1.67)	0	0	8 (10.13)
Total (%)	107 (44.77)	194	29 (14.95)	119 (61.34)	34 (17.53)	12 (6.19)	118 (60.82)	103 (51.55)	87 (44.85)	60 (30.93)	29 (14.95)	26 (13.40)	79 (40.72)

(^*^) distribution of *L. monocytogenes* serotypes/genetic determinants of virulence in RTE foods is not statistically significant (p > 0.01).

(LIPI-1) isolates harbouring all LIPI-1 virulence genes.

*inl* (A, B, C, J) isolates harbouring all the internalin genes.

LIPI-1 + *inl* (A, B, C, J) isolates that harbour all the LIPI and the internalin genes.

(NT) Not serotyped.

### Genotypic determinants and virulence of *L. monocytogenes* isolates recovered from ready RTE foods

#### Serotypes

**Figure 1:**
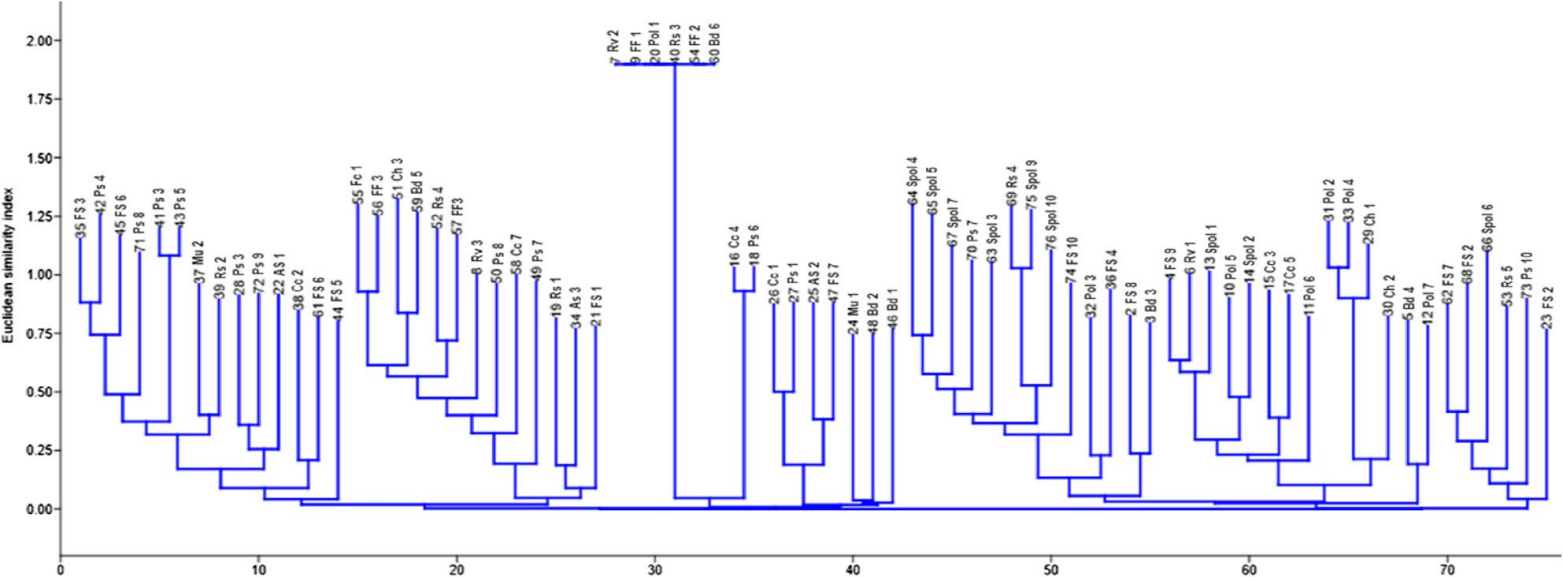
A neighbour-joining dendrogram of ERIC-PCR fingerprints of *L. monocytogenes* strains indicates similarities between clustered isolates and their sources. Pol, polony; Spol, sliced polony; FS, fruit salad; Ch, chips; FF, fried fish; Rs, Russian sausage; Rv, red Vienna; Bd, bread; Fc, fried chicken; Vk, vetkoek; Mps, meat pie; Cc, cupcakes; Mu, muffins; As, assorted sausages.

Lm was classified into four (4) distinct epidemiological groups (1/2a, 1/2b, 1/2c and 4b) based on the PCR procedure described by^22^. Three Lm serotypes were identified among 194 Lm isolates belonging to serotypes (1/2a, 1/2b and 4b), as presented in (Table 2). The 1/2b serotype, (n = 119, 61.34%) was the most prevalent. The highest prevalence of Serotype 1/2b was observed in fruit salad and sliced polony, others were randomly distributed across the food matrices. Serotype 4b (n = 34, 17.53%) isolates were detected in all food samples except fried chicken while serotype 1/2a (n = 29, 14.95%) was distributed across other RTE food matrices at various percentiles but was not detected in Vienna sausage, bread, and fried chicken. Figure 1 represent the gel image of the PCR amplification of Lm serotypes.

#### Virulence genes

Virulence genes play significant roles in the pathogenicity of Lm^12^. The prevalence of 10 virulence genes assessed revealed that *inl*A*, Inl*C, *inl*J, *plc*A, *Prf*A were detected in all Lm isolates; *hly*A (n = 180, 92.78%)*,* *plc*B (n = 176, 90.72%), *mpl, inl*B (n = 167, 86.08%)*, *and *act*A (n = 148, 76.29%) varies (Fig. S3). Table 2 describes the genotypic virulence profile of the listeriosis agent recovered from RTE food samples. All the LIPI + *inl* (A, B, C and J) virulence gene clusters were detected in all RTE foods (n = 87, 44.85%), and the highest prevalence was observed among isolates from fruit salad (n = 24, 27.59%) followed by (n = 11, 12.64%) for polony, sliced polony and potato chips respectively. The *inl*A, B, C and J were detected among 103 (53.09%) Lm isolates recovered from RTE foods while the LIPI-1 virulence gene cluster was detected among 118 (60.82%) of the RTE food isolates. The *inl*A, B, C and J were detected among 103 (53.09%) Lm isolates recovered from RTE foods while the LIPI-1 virulence gene cluster was detected among 118 (60.82%) of the RTE food isolates. The Spearman’s correlation coefficient (rho) revealed that the virulence genes that constitute the LIPI-1 (rho = 0.967), the internalin A, B, C and J (rho = 0.981), and the combination of the LIPI-1 + *inl* (A, B, C, and J) (rho = 0.964) were significantly (p < 0.01) associated with the RTE food isolates. The data obtained were further subjected to a Chi-square test to ascertain the frequencies and association of serotypes and genetic determinants of virulence in RTE foods (Table 2). The highest prevalence of serotype 1/2a was observed in pie, serotypes 1/2b and 4b were observed in fruit salad, while isolates that were not typed were more prevalent in pies. The Chi-square test revealed no significant differences (p > 0.01) in the prevalence of serotype 1/2a, 1/2b and 4b and the LIPI-1, *inl* (A, B, C and J) and LIPI-1 + *inl* (A, B, C and J) in all the RTE foods analyzed.

### Adherence and biofilm-forming potential

The RTE food isolates were tested for biofilm-forming, and the analysis revealed that 134 (69.07%) were potential biofilm formers. The biofilm-forming ability were classified as weak (n = 29, 14.95%), moderate (n = 26, 13.40%) and strong (n = 79, 40.72%). Furthermore, the data were analyzed to determine the association of the isolate’s biofilm-forming capabilities with specific RTE foods. Spearman’s correlation revealed significant association (p ≤ 0.01) between Lm biofilm forming Lm and fruit salad (rho = 0.774), chips (rho = 0.760), Russian sausage (0.926), meat pie and polony (rho = 1.000) and cupcakes (0.926) at (p ≤ 0.01) while sliced polony (rho = 0.518) and fried fish (rho = 0.598) were statistically significant at (p ≤ 0.05). The biofilm-forming potentials of the serotypes observed revealed that the most of 1/2a (n = 10, 34.48), 1/2b (n = 54, 45.38) and 4b (n = 13, 38.24%) were strong biofilm formers (Table 3).

**Table 3:** Biofilm forming capability of the serotypes observed.

Serotype	Biofilm formation (%)				Total
	Negative	Weak	Moderate	Strong	
1/2a	8 (27.59)	7 (24.14)	4 (13.79)	10 (34.48)	29 (14.95)
1/2b	31 (26.05)	17 (14.29)	17 (14.29)	54 (45.38)	119 (61.34)
4b	14 (41.18)	4 (11.76)	3 (8.82)	13 (38.24)	34 (17.53)
Not serotyped	7 (58.33)	1 (8.33)	2 (16.67)	2 (16.67)	12 (6.19)
Total (%)	60 (30.93)	29 (14.95)	26 (13.40)	79 (40.72)	194 (100)

### Intra-species multiplicity of *L. monocytogenes* isolates

Lm isolates recovered from food matrices (polony, sliced polonies, fruit salad, Russian sausages, assorted sausages, muffins, cupcakes, pies, chips, bread, Vienna sausages, fried fish and fried chicken isolates) were analyzed to determine their genetic relatedness/diversities. The ERIC-PCR fingerprints were employed for the classification of the genetic relatedness of the isolates by the dendrogram produced (Fig. S3) by Paleontological Statistical software version 4.03 (PAST 4.03). The dendrogram was clustered into eight clades of Lm strains numbering from the origin (0) along the x-axis.

## Discussion

* Listeria monocytogenes* (Lm) is an important pathogenic microorganism recognized for listeriosis outbreaks^12^. This makes it imperative to conduct an epidemiological assessment of foods that could be of high risk and pose health threats to consumers. Few studies had earlier reported the prevalence of Lm in RTE foods in RSA^38^. Although the presumptive Lm plate counts observed in our study were relatively low, the presence of the pathogen is still a potential health risk factor to consumers considering the ability of Lm growth in food during preservation and storage. About 30.54% of the RTE food samples recorded counts ranging between 1 and 10 CFU/g, 10.46% for counts ranging between 10 and 100 CFU/g and 3.77% recorded counts above the 100 CFU/g benchmarks. In other words, 96.77% of the RTE food met the 100 CFU/g limits adopted by the EU states for foods and 3.77% failed to meet the EU benchmark. Previous studies by EFSA on risk assessment suggested that most invasive cases (over 90%) of listeriosis could be caused by ingesting RTE foods laden with > 2000 CFU/g, and about 33% of cases could be due to Lm growth in foods at the consumer or storage phase^39^. Significant (p < 0.01) contamination was observed in specific RTE foods including sliced polony, potato chips, assorted sandwiches, Russian sausages, fruit salad, bread, pie, polony, and cupcake. This suggests that these foods could be at higher risk of Lm contamination among the RTE food samples analyzed. Also, Lm virulence varies considerably by strain and the susceptibility of the individual at risk of infection could also vary^40^. However, the exact estimate of Lm infectious dose that could induce infection is not yet known^12^. Our study revealed that a larger proportion (55.23%) met the zero tolerance for the detection of Lm in RTE foods whereas, (44.77%) failed the zero-tolerance level adopted in the USA. A previous study documented the detection of Lm from processed delicatessens (sandwiches, salads, desserts and sausages), meat and fish products^7^. The prevalence recorded in our study is way higher than the 25% detection of Lm in RTE foods and meats reported in Chile^41^, 17.70% reported in RTE foods in Poland^7^ and 14.7% observed in meat and meat products in South Africa^38^. Of note, Russian sausage (78.57) and sliced polony (61.90) were two contaminated RTE food products with the highest prevalence of Lm. The prevalence of Lm in RTE foods in this study is at variance with that previously reported by Montero et al. in some RTE foods such as cheese, pate, sausages and shellfish (40, 55, 13 and 25%) respectively^41^. This could be attributed to unhygienic handlings/practices or a high level of exposure to contaminants from humans, surfaces of processing facilities and cross-contamination after processing. It could also be due to the ability of Lm to proliferate during the storage of the RTE foods at refrigeration^41^.

Serotyping Lm is vital in epidemiological surveillance, especially in food safety assessment and monitoring^42^. Strains of Lm belonging to serotypes 1/2a, 1/2b, 1/2c and 4b are mostly found in cases of listeriosis in humans^12,23,41^. Three serotypes were detected among Lm recovered from RTE foods including the 1/2a (14.95%), 4b (17.53%) serotypes, and 1/2b (61.34%) being the highest prevalence observed. A similar trend was observed in the study of Chen et al. on fresh aquatic products in China^23^. A previous study^43^ observed that about 87% of Lm strains recovered from food in Montevideo-Uruguay belong to serotypes 1/2b (33, 45.83%), 4b (30, 41.67%), 1/2a (5, 6.94%) and 1/2c (3, 4.17%). Similarly, serotype 1/2a (23%), serotype 1/2b (25%) and serotype 4b (52%) of Lm were isolated from listeriosis cases in Chile^41^. The high prevalence of serotypes 1/2b and 4b in our study could be attributed to the higher genetic variation observed in lineage II strains in consonance with adaptation to diverse environments^43–45^. Serotype 1/2b and 4b prevalence observed in RTE foods in our study were higher while serotype 1/2a was lower compared with the prevalence described by Chen et al. in meat and meat products in China^46^. In contrast, (66%) prevalence of serotype 4b was documented for seafood and (100%) for cheese^41^ was higher than the prevalence of serotype 4b in RTE foods in our study. Another study reported a significant (p < 0.05) association of serotype 1/2b with cooked sausage, smoked fish 1/2a with pate, and serotype 1/2c with raw meat but no association with serotype 1/2b and fresh vegetables^41^. Also, the report from a previous study conducted in China indicated serogroup I.1 (1/2a, 38.89%), II.2 (1/2b, 34.72), and serogroup (4c, 22.22%) were the predominant Lm serotypes whereas, serogroup II.1 (4b) was detected at a lower frequency^23^. Furthermore, the predominance of serotypes 1/2b (45.4%), 1/2a (33.3%) and 1/2c (14.2%) were reported in RTE foods in Zigong, China^47^. The association of these strains with RTE foods observed in our report calls for the need for regular assessment of RTE foods as most of the foods is rarely heated before consumption. More importantly, preventive measures are needed to be enforced to guarantee food safety for consumers in the Republic of South Africa.

Virulence genes play a significant role in the pathogenicity expressed by Lm and may be responsible for the health challenges that manifest after foods contaminated with potentially pathogenic strains are ingested^41^. These genes, especially those found in the LIPI-1 regulate the virulence transcription of *prf*A, and other virulence factors involved in stages of intracellular infections. The phospholipases (phosphatidylinositol—*plc*A and phosphatidylcholine—*plc*B) enhance the lysis of the host cellular membranes whereas the listeriolysin (*hly*) ensures bacterial evasion from host cells. Metalloproteases are required for the extracellular activations and the maturation of *plc* B virulence gene. The *act* A gene combined action with *mpl* and *plc * B are required for cell-to-cell spread ^12^. In addition, specific sub-lineage pathogenicity islands, like the LIPI-3 and LIPI-4 encoding the bacteriocin LLS and a putative cellobiose family phosphotransferase system, respectively, promote bacterial colonization and enhance neurovirulence when present in combination with LIPI-I. Ten virulence genes including the *inl*A*, Inl*C, *inl*J, *plc*A, *prf*A*, hly*A*,* *plc*B, *mpl inl*B*,* and *act*A were detected by PCR assays at varying proportions among Lm isolates in our study. The internalin genes were more frequently detected among the isolates recovered from RTE foods. All the LIPI-1 + *inl* (A, B, C and J) virulence gene clusters were detected in RTE foods isolates, and the highest prevalence was observed among isolates from fruit salad followed by polony, sliced polony and potato chips respectively. All the *inl*A, B, C and J were detected among 53.09% of Lm isolates recovered from RTE foods while the LIPI-1 virulence gene cluster was detected among 60.82% of the RTE food isolates. This is similar to the documentation of^41^ who reported one or more LIPI-1 genes detected in all strains isolated from RTE foods in Chile. Du et al. reported the presence of the *Inl*A, *inl*C*, inl*J, *hly*A, *plc*B and *prf*A in all isolates while *inl*B, *act*A, and *plc*A were found in 71.4–90.5% of the isolates recovered from RTE foods in China^35^ similar to the trends observed in our report. In contrast, *Inl*A, *inl*B, *prf*A, *plc*B, *act*A, *hly, iap, mpl* and *plc*B was reported in all isolates recovered from aquatic products in China^23^. Furthermore, Nielsen and colleagues detected virulent markers among 95% of the isolates using the whole genome sequencing (WGS) technique^48^. The internalin (*inl*A, B, C, and J) is a group of well-studied virulence markers due to the critical role it plays in mediating the internalization and adhesion of Lm inside the epithelial cell^41^. The high prevalence rate of virulence genes reported in this study is similar to those documented in previous studies^23,35,49^. In contrast, *inl* (A, B, C and J) was detected in all strains of Lm recovered from RTE foods in China^50^. The virulence genes that constituted the LIPI-1, the internalin A, B, C, and J and the LIPI-1 + *inl* (A, B, C, and J) were strongly (p < 0.01) associated with the RTE food isolates. Furthermore, the Chi-square test revealed no significant association (p > 0.01) in the association of serotype 1/2a, 1/2b and 4b and the LIPI-1, *inl* (A, B, C and J) and LIPI-1 + *inl* (A, B, C and J) in all the RTE foods analyzed. A previous study^41^ described the prevalence of virulence genes to be more associated with serotype 4b and less frequent as obtained for 1/2a and 1/2c serotypes. The prevalence and association of these genes with the epidemiological Lm strains in our study could project the isolates as a potential threat to public health, especially to consumers of such foods. However, the existence of virulence genes in the isolates might not necessarily provide sufficient proof that they could trigger the infection process in humans^41^. An experimental report had previously revealed Lm strains with low pathogenic potential were found harbouring such genes^51^. Conversely, Roche et al.^52^ documented that pathogenic strains which demonstrated highly virulent profiles in vitro were not found to demonstrate virulence in animal models.

Most of the Lm isolates demonstrated the potential for biofilm formation and were categorized as weak moderate and strong biofilm formers (Tables 2 and 3). It is necessary to consider the relationship between Lm serotypes and the potential for biofilm formation. Most of the isolates tested for biofilm potential in this study belong to serotypes 1/2a, 1/2b and 4b. Higher biofilm-forming potential was observed among serotype 1/2b (61.34%) and 4b (17.53), compared with serotype 1/2a (14.95). Lm serogroups differ in their prevalence and ecological distribution. The epidemiological strains frequently associated with food and cases of human listeriosis are members of the serotypes 1/2a, 1/2c, 1/2b and 4b^41^. This could likely suggest why higher biofilm formation was observed among serotype 1/2b isolates. More importantly, most of the isolates recovered from chips, fried fish, polony and fruit salad are strong biofilm formers. As such, the Lm strain that was implicated in the last listeriosis outbreak recorded in the Republic of South Africa may possess a biofilm-forming potential that possibly enhanced their persistent presence in polony, and similarly in other RTE foods. Some scholars have earlier documented that there exists a relationship between the strains of the serotypes 1/2a, 1/2c, and 1/2b in the food processing context and their capacity for greater biofilm-forming potential^53–56^. The ability of Lm to form biofilm is one important characteristic/mechanism used to resist the effect of sanitisers and disinfectants applied to food processing contact surfaces^57^. This has possibly escalated the frequent isolation of this pathogen in processed foods and further compromised food safety for human consumption.

Source tracking of Lm is an important technique that has been employed in the epidemiological surveillance of infectious agents as it reveals the genetic relatedness and variability of Lm strains due to the ecological diversity of the pathogen^41,42^. Methods include whole-genome sequencing (WGS)^48^, multilocus sequence typing (MLST), ERIC-PCR (enterobacterial repetitive intergenic consensus), gene sequencing, PCR sequencing, and PCR ribotyping^42^. Although pulsed-field gel electrophoresis (PFGE) is usually regarded as the gold standard for genotyping due to its high reproducibility and discriminatory strength^41,42^, it is more expensive and labour intensive whereas, ERIC-PCR is easy, cheaper, faster and also dependable^58^. In this study, ERIC-PCR typing has demonstrated simplicity, reproducibility, reliability, and adaptability in differentiating/classifying strains of microbial species based on their genetic relatedness and intra-species diversity. The molecular typing technique (ERIC PCR) used in this study revealed high genetic diversity among Lm isolates studied. The DNA fingerprints generated enhanced the comparison of the DNA patterns and their classification into clusters based on their similarities. The dendrogram produced by the computer-assisted analysis generated 8 clades. This is an indication of the high genetic diversity of the RTE food isolates recovered from different sources (Fig. 1). Isolates from different RTE foods forming clusters are proof of the evolutionary relationship amongst the isolates. The genetic diversity observed among isolates obtained from RTE foods from different sources revealed the relevance of this typing technique in epidemiological outbreak investigation and surveillance. The association of these epidemiological strains with RTE foods does not necessarily identify such foods as a transmission vehicle for Lm. Sequence type 6 (serotype 4b) was identified as the major player in the listeriosis outbreak a few years back in the Republic of South Africa^59^. This study characterized serotype 4b and other epidemiological strains belonging to serotypes 1/2a and 1/2b that were frequently identified in listeriosis outbreaks, thus building the epidemiological history of Lm in the Republic of South Africa.

## Conclusion

This study revealed the prevalence, virulence signatures, biofilm and genetic diversity of Lm recovered from RTE foods obtained from different retail outlets in various locations in South Africa. Based on the epidemiological history of Lm linked to several foodborne outbreaks in several instances globally, the virulence status of the Lm strains isolated in these food matrixes revealed that the isolates could constitute potential health threats (listeriosis), especially among immunocompromised individuals (including HIV and tuberculosis and diabetic patients) in SA. Furthermore, the prevalence and contamination levels of RTE foods compromised food safety for human consumption. We, therefore, recommend that adequate governmental legislation and monitoring programs should be implemented with adequate enforcement. Also, proper regulation of the food sector must be ensured to avoid any future health crisis that could be linked to a (listeriosis) foodborne outbreak in the Republic of South Africa.

## Supplementary Information

Supplementary Information 1.

Supplementary Information 2.

## Data Availability

All data generated or analysed during this study are included in this published article and its supplementary information files (RTE food isolates raw data).
