# Supplementary material for: Assessment of the molecular epidemiology and genetic multiplicity of Listeria monocytogenes recovered from ready-to-eat foods following the South African listeriosis outbreak
Source: Sci Rep. 2022 Nov 22;12:20129. doi: 10.1038/s41598-022-20175-x (PMC9684121; doi:10.1038/s41598-022-20175-x)
Supplement: Supplementary file 1 — Supplementary Information 1. [file 41598_2022_20175_MOESM1_ESM.pdf]

Biofilm raw data

Isolates no	Absorbance	Average absorbance	Biofilm classification	serotype	Isolates source
1	0.522	0.467	Weak	nt	sp
	0.533				
	0.346				
2	0.82	0.602667	Weak	nt	sp
	0.465				
	0.523				
3	0.589	0.636	Weak	2a	sp
	0.573				
	0.746				
4	0.467	0.346333	Negative	2a	sp
	0.266				
	0.306				
5	0.332	0.355333	Negative	nt	sp
	0.345				
	0.389				
6	0.386	0.324667	Negative	nt	sp
	0.181				
	0.407				
1	0.698	0.611	Weak	nt	Rs
	0.544				
	0.591				
1	0.331	0.360667	Negative	nt	ps
	0.393				
	0.358				
2	0.327	0.371333	Negative	nt	ps
	0.391				
	0.396				
3	0.448	0.423333	Weak	nt	ps
	0.361				
	0.461				
4	0.343	0.384333	Negative	nt	ps
	0.354				
	0.456				
1	0.445	0.438	Negative	nt	fs
	0.515				
	0.354				
7	0.423	0.424667	Weak	4b	sp
	0.461				
	0.39				
8	0.396	0.424667	Weak	4b	Sp

	0.41				
	0.468				
9	0.43	0.505333	Weak	nt	Sp
	0.54				
	0.546				
10	0.785	0.566667	Weak	nt	Sp
	0.43				
	0.485				
5	0.39	0.428	Weak	2b	ps
	0.448				
	0.446				
6	0.333	0.467333	Weak	nt	ps
	0.671				
	0.398				
7	0.527	0.463	Weak	2b	ps
	0.536				
	0.326				
2	0.75	0.609	Weak	2b	rs
	0.797				
	0.28				
1	0.389	0.686667	Weak	2b	fc
	0.474				
	1.197				
2	0.501	0.512	Weak	2b	fc
	0.498				
	0.537				
1	0.465	0.491667	Weak	2b	ch
	0.541				
	0.469				
2	1.116	0.855667	Moderate	2b	ch
	0.779				
	0.672				
3	0.45	0.653	Weak	2b	ch
	0.589				
	0.92				
11	0.503	0.507333	Weak	2b	sp
	0.685				
	0.334				
12	0.347	0.371333	Negative	2b	sp
	0.483				
	0.284				
133	0.881	0.835667	Moderate	2b	sp
	0.829				

	0.797				
14	0.472	0.533333	Weak	2b	sp
	0.928				
	0.2				
15	0.472	563	Weak	2b	sp
	0.601				
	0.616				
1	0.579	480667	Weak	nt	muffins
	0.315				
	0.548				
2	0.982	1.035667	Moderate	2b	muffins
	1.066				
	1.059				
1	0.904	1.024	Moderate	2b	ff
	1.056				
	1.112				
2	0.802	706	Weak	2b	ff
	0.66				
	0.656				
3	0.689	729333	Weak	2b	ff
	0.608				
	0.891				
4	0.831	0.756667	Weak	2b	ff
	0.708				
	0.731				
4	0.915	868	Moderate	2b	ch
	0.782				
	0.907				
5	0.779	0.808667	Moderate	2b	Ch
	0.745				
	0.902				
6	0.581	0.698333	Weak	2b	Ch
	0.593				
	0.921				
5	0.777	0.721333	Weak	2b	ff
	0.494				
	0.893				
6	0.971	0.976	Moderate	2b	ff
	0.978				
	0.979				
7	0.758	0.836667	Moderate	nt	ff
	0.893				
	0.859				

1	0.773	0.764667	Weak	2b	p
	0.75				
	0.771				
1	1.453	1.54733	Moderate	nt	Cc
	1.492				
	1.697				
7	1.212	1.180333	Moderate	2b	ch
	1.243				
	1.086				
3	0.87	1.038333	Moderate	2b	rs
	1.093				
	1.152				
1	0.734	0.709	Weak	nt	bread
	0.763				
	0.63				
2	0.773	0.75	Weak	2b	P
	0.759				
	0.718				
3	0.825	0.795	Weak	2b	P
	0.73				
	0.83				
16	0.935	0.930667	Moderate	2b	sp
	0.858				
	0.999				
17	0.821	0.754333	Weak	2b	sp
	0.734				
	0.708				
18	1.556	1.291667	Moderate	2b	sp
	1.221				
	1.098				
19	1.11	1.014667	Moderate	2b	sp
	0.97				
	0.964				
2	0.717	0.827333	Moderate	2b	Cc
	0.908				
	0.857				
8	1.13	1.033667	Moderate	nt	ch
	1.103				
	0.868				
9	1.596	1.58233	Moderate	2b	ch
	1.632				
	1.519				
4	1.031	1.143333	Moderate	2b	rs

	1.313				
	1.086				
4	0.995	1.033	Moderate	2b	p
	1.147				
	0.957				
2	0.738	0.604	Weak	2b	fs
	0.544				
	0.53				
3	0.726	0.662	Weak	nt	muffins
	0.723				
	0.537				
1194	0.661	0.744333	Weak		
	0.775				
	0.797				
1	0.788	0.754667	Weak	2b	As
	0.812				
	0.664				
2	0.941	0.901667	Moderate	nt	As
	0.894				
	0.87				
3	2.835	2.232333	Strong	2b	As
	2.078				
	1.784				
3	1.981	2.106667	Strong	2b	fs
	1.932				
	2.407				
4	1.497	1.581333	Moderate	2b	fs
	1.197				
	2.05				
4	1.715	1.861667	Strong	2b	muffins
	1.942				
	1.928				
5	1.194	1.22	Moderate	2b	muffins
	1.224				
	1.242				
6	0.996	0.947333	Moderate	2b	muffins
	0.928				
	0.918				
4	1.099	1.438667	Moderate	2b	As
	1.034				
	2.183				
5	0.833	0.934333	Moderate	2b	As
	0.947				

	1.023				
6	0.692	0.916	Moderate	2b	As
	0.949				
	1.107				
7	0.819	0.694	Weak	2b	As
	1.084				
	0.989				
8	1.08	1.066667	Moderate	2b	As
	0.848				
	1.272				
10	1.041	1.215667	Moderate	2b	Ch
	1.082				
	1.524				
5	1.024	1.293	Moderate	2b	Rs
	1.097				
	1.758				
6	1.145	1.083333	Moderate	2b	Rs
	1.044				
	1.061				
8	1.014	1.217333	Moderate	2b	Ps
	1.104				
	1.534				
9	1.269	1.283	Moderate	2b	Ps
	1.085				
	1.495				
5	1.083	1.213667	Moderate	2b	Fs
	1.338				
	1.22				
6	1.028	1.166	Moderate	2b	Fs
	1.162				
	1.308				
10	0.793	0.998667	Moderate	nt	Ps
	1.017				
	1.186				
11	1.075	1.25333	Moderate	2b	Ch
	1.227				
	1.458				
12	1.235	1.212667	Moderate	2b	Ch
	1.348				
	1.055				
13	1.275	1.163333	Moderate	2b	Ch
	1.111				
	1.104				

14	1.418	1.407667	Moderate	2b	Ch
	1.383				
	1.422				
15	1.108	1.363333	Moderate	2b	Ch
	1.388				
	1.594				
16	1.381	1.450333	Moderate	nt	Ch
	1.493				
	1.477				
5	1.25	1.170333	Strong	4b	P
	1.173				
	1.088				
6	1.309	1.309333	Moderate	4b	P
	1.226				
	1.393				
7	1.988	1.941	Strong	2b	P
	1.805				
	2.03				
8	0.475	0.415667	Medium	2b	P
	0.408				
	0.364				
9	0.382	0.484333	Medium	2b	P
	0.561				
	0.51				
10	0.574	0.442	Medium	2b	P
	0.361				
	0.391				
11	0.368	0.566667	Strong	2b	P
	0.387				
	0.945				
12	0.369	0.387333	Medium	2b	P
	0.372				
	0.421				
13	0.347	0.35333	Medium	2b	P
	0.341				
	0.372				
9	0.31	0.405333	Medium	2b	As
	0.475				
	0.431				
7	0.69	0.826333	Strong	2b	Fs
	1.136				
	0.653				
8	0.642	0.67	Strong	2b	Fs

	0.681				
	0.687				
9	0.379	0.468667	Medium	2b	Fs
	0.53				
	0.497				
10	0.66	0.581	Strong	2b	Fs
	0.5				
	0.583				
11	0.751	0.581	Strong	nt	Fs
	0.855				
	0.959				
12	0.378	0.390333	Medium	nt	Fs
	0.376				
	0.417				
7	0.404	0.392667	Medium	2b	muffins
	0.412				
	0.362				
8	0.357	0.401333	Medium	2b	muffins
	0.388				
	0.459				
9	0.491	0.770667	Strong	nt	muffins
	0.959				
	0.862				
10	0.911	0.813333	Strong	2b	mufffins
	0.796				
	0.733				
3	0.738	0.612	Strong	2b	Cup cakes
	0.598				
	0.5				
7	0.615	0.501333	Medium	2b	Rs
	0.451				
	0.438				
8	0.915	0.664333	Strong	2b	Rs
	0.583				
	0.495				
9	0.748	0.819	Strong	2b	Rs
	0.89				
	0.819				
10	0.589	0.502333	Medium	2b	Rs
	0.469				
	0.449				
10	0.411	0.387667	Medium	2b	Ps
	0.345				

	0.407				
11	0.368	0.493	Medium	2b	Ps
	0.66				
	0.451				
12	0.825	0.796333	Strong	2b	Ps
	0.768				
	0.796				
13	0.721	0.796333	Strong	nt	Ps
	0.742				
	0.64				
14	0.765	0.68	Strong	nt	Ps
	0.649				
	0.626				
15	1.226	0.858	Strong	2b	Ps
	0.735				
	0.613				
8	1.681	1.03333	Strong	2b	Ff
	0.745				
	0.674				
13	0.635	0.674	Strong	2b	Fs
	0.654				
	0.733				
14	0.745	0.973667	Strong	2b	Fs
	0.92				
	1.256				
15	0.878	0.994233	Strong	2b	Fs
	1.066				
	0.883				
16	0.469	0.404	Medium	2b	Fs
	0.373				
	0.37				
17	0.396	0.372	Medium	2b	Fs
	0.338				
	0.382				
18	0.622	0.684333	Strong	2b	Fs
	0.755				
	0.676				
2	0.498	0.454333	Medium	nt	bread
	0.411				
	0.454				
3	0.447	0.383	Medium	2b	bread
	0.353				
	0.349				

19	0.332	0.355	Medium	2b	Fs
	0.367				
	0.366				
20	0.405	0.417	Medium	2b	Fs
	0.458				
	0.388				
4	0.343	0.542333	Strong	2b	bread
	0.722				
	0.562				
5	0.541	0.518333	Strong	2b	bread
	0.511				
	0.503				
21	0.938	0.897667	Strong	2b	Fs
	0.835				
	0.92				
22	0.68	0.834667	Strong	2b	Fs
	0.896				
	0.928				
6	0.748	0.686667	Strong	2b	bread
	0.72				
	0.592				
7	0.571	0.652	Strong	2b	bread
	0.659				
	0.726				
23	0.851	0.838333	Strong	2b	Fs
	0.799				
	0.865				
24	0.614	0.548667	Medium	2b	Fs
	0.474				
	0.558				
25	0.413	0.404	Medium	2b	Fs
	0.369				
	0.43				
26	0.341	0.471333	Medium	2b	Fs
	0.469				
	0.604				
8	0.505	0.479667	Medium	2b	bread
	0.393				
	0.541				
1	0.528	0.494	Medium	2b	Rv
	0.483				
	0.471				
2	0.457	0.366333	Medium	2b	Rv

	0.391				
	0.251				
3	0.622	0.618333	Strong	2b	Rv
	0.603				
	0.63				
4	0.65	0.689667	Strong	2b	Rv
	0.64				
	0.779				
9	0.892	0.689667	Strong	2b	Ff
	0.763				
	0.583				
14	0.484	0.642667	Strong	n	P
	0.522				
	0.922				
15	0.643	0.71	Strong	n	P
	0.766				
	0.721				
16	0.776	0.672	Strong	2b	P
	0.581				
	0.659				
17	0.777	0.688	Strong	n	P
	0.716				
	0.571				
18	0.657	0.617	Strong	2b	P
	0.685				
	0.509				
19	2.321	1.207	Strong	n	P
	0.848				
	0.452				
20	0.467	0.443667	Medium	2b	P
	0.374				
	0.49				
20	0.525	0.544667	Medium	2b	Sp
	0.651				
	0.458				
21	0.39	0.429667	Medium	n	Sp
	0.465				
	0.434				
22	0.819	0.572	Medium	n	Sp
	0.467				
	0.43				
23	0.338	0.557667	Medium	2b	Sp
	0.98				

	0.355				
4	0.739	0.774667	Strong	n	Cc
	0.743				
	0.842				
5	1.007	0.689	Strong	4b	Cc
	0.704				
	0.356				
6	0.57	0.622	Strong	2b	Cc
	0.623				
	0.673				
7	0.606	0.669667	Strong	4b	Cc
	0.662				
	0.741				
8	0.791	0.716333	Strong	2b	Cc
	0.67				
	0.688				
9	0.585	0.453333	Medium	2b	Cc
	0.375				
	0.4				
16	0.715	0.587	Strong	2b	Ps
	0.537				
	0.509				
17	0.443	0.456667	Medium	2b	Ps
	0.479				
	0.448				
18	0.516	0.644	Strong	2b	Ps
	0.817				
	0.599				
19	0.56	0.489	Medium	n	Ps
	0.513				
	0.394				
20	0.412	0.379333	Medium	n	Ps
	0.353				
	0.373				
21	0.767	0.695667	Strong	n	Ps
	0.671				
	0.649				
22	0.651	0.618667	Strong	n	Ps
	0.61				
	0.595				
20	0.501	0.462	Medium	n	ff
	0.364				
	0.521				

11	0.476	0.512333	Medium	n	Rs
	0.436				
	0.625				
12	0.557	0.535333	Medium	n	Rs
	0.569				
	0.48				
13	0.408	0.408333	Medium	n	Rs
	0.425				
	0.392				
14	0.384	0.344	Medium	2b	Rs
	0.318				
	0.33				
10	0.469	0.514	Medium	n	Ff
	0.491				
	0.582				
11	0.427	0.434333	Medium	2b	Ff
	0.498				
	0.378				
13	0.564	0.389	Medium	n	Ff
	0.309				
	0.294				
14	0.252	0.274	Medium	2b	Ff
	0.284				
	0.286				
15	1.031	0.642667	Strong	2b	Ff
	0.476				
	0.421				
16	0.358	0.336333	Medium	n	Ff
	0.338				
	0.313				
17	0.331	0.313	Medium	2b	Ff
	0.295				
	0.313				
18	0.386	0.418333	Medium	2b	Ff
	0.404				
	0.465				
19	1.185	0.731667	Strong	2b	Ff
	0.731				
	0.279				
10	0.506	0.468333	Medium	2b	Cc
	0.409				
	0.49				
11	0.656	0.497333	Medium	2b	Cc

	0.367				
	0.469				
12	0.566	0.556333	Medium	2b	Cc
	0.471				
	0.632				
9	0.444	0.297	Medium	2b	bread
	0.22				
	0.227				
10	0.339	0.257333	Weak	2b	bread
	0.242				
	0.191				
11	0.286	0.168	Weak	2b	bread
	0.089				
	0.129				
27	0.125	0.239333	Weak	2b	fs
	0.268				
	0.325				
28	0.236	0.216333	Weak	2b	fs
	0.225				
	0.188				
29	0.31	0.278667	Medium	2b	fs
	0.262				
	0.264				
30	0.055	0.138	Negative	2b	fs
	0.22				
	0.139				
11	0.169	0.138	Negative	2b	muffins
	0.112				
	0.133				
12	0.287	0.248333	Weak	2b	muffins
	0.221				
	0.237				
Negative control	0.497	0.401667	negative		
	0.403				
	0.305				
Positive control Lm1	0.976	1.208667			
	1.351				
	1.299				

Pol – polony, Spol - sliced polony, FS - fruit salad, Ch - chips, FF - fried fish, Rs - Russian sausage, Rv - red vienna, Bd – bread, Fc - fried chicken, Vk - vetkoek, Mps – pie, Cc – cupcakes, Mu - muffins, As - assorted sausages

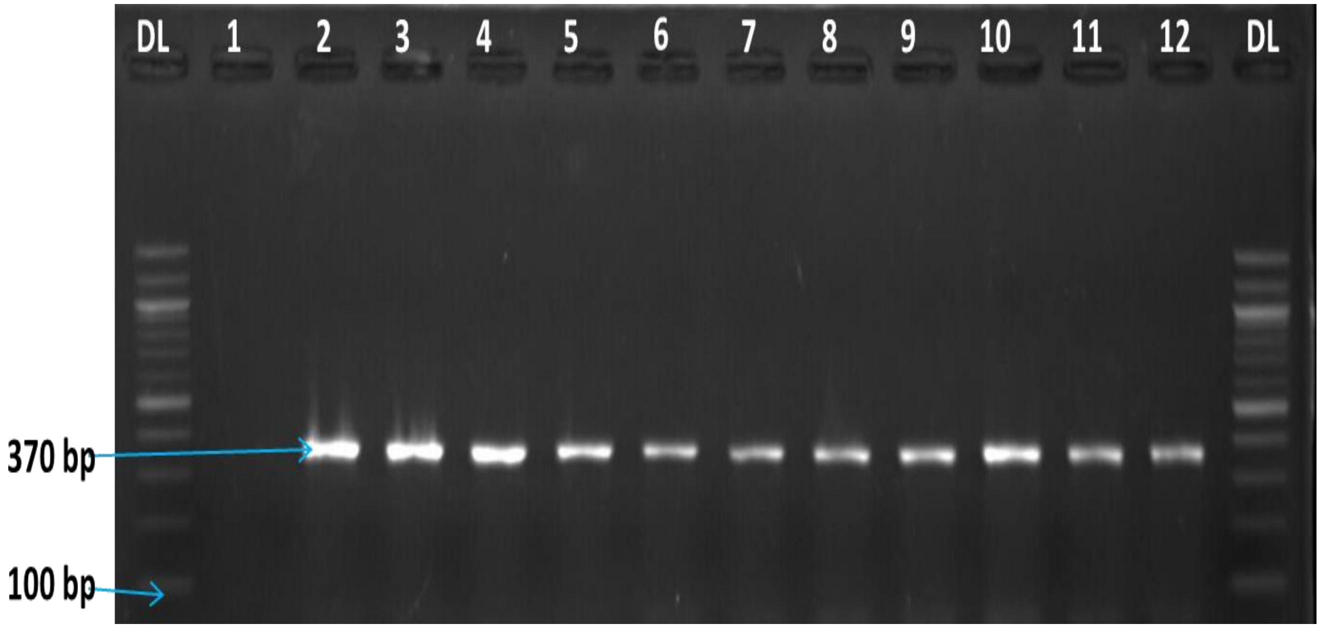

prs gene raw gel for the amplification of *Listeria* genus

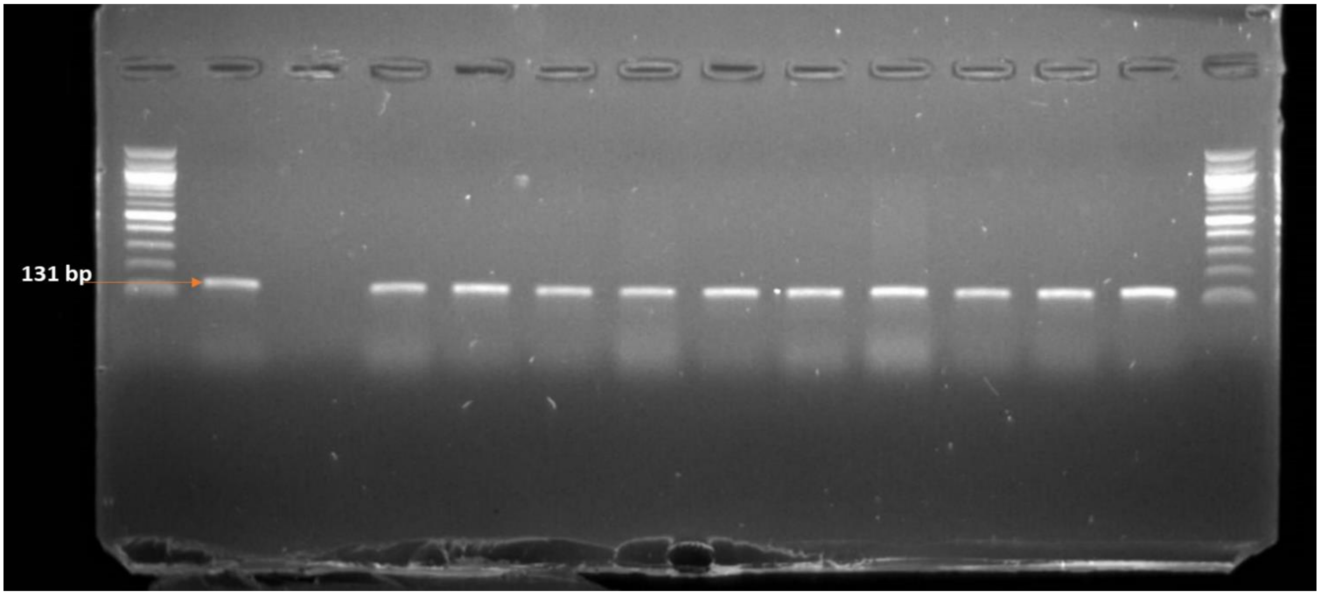

iap gene raw gel for the amplification of *L. monocytogenes*

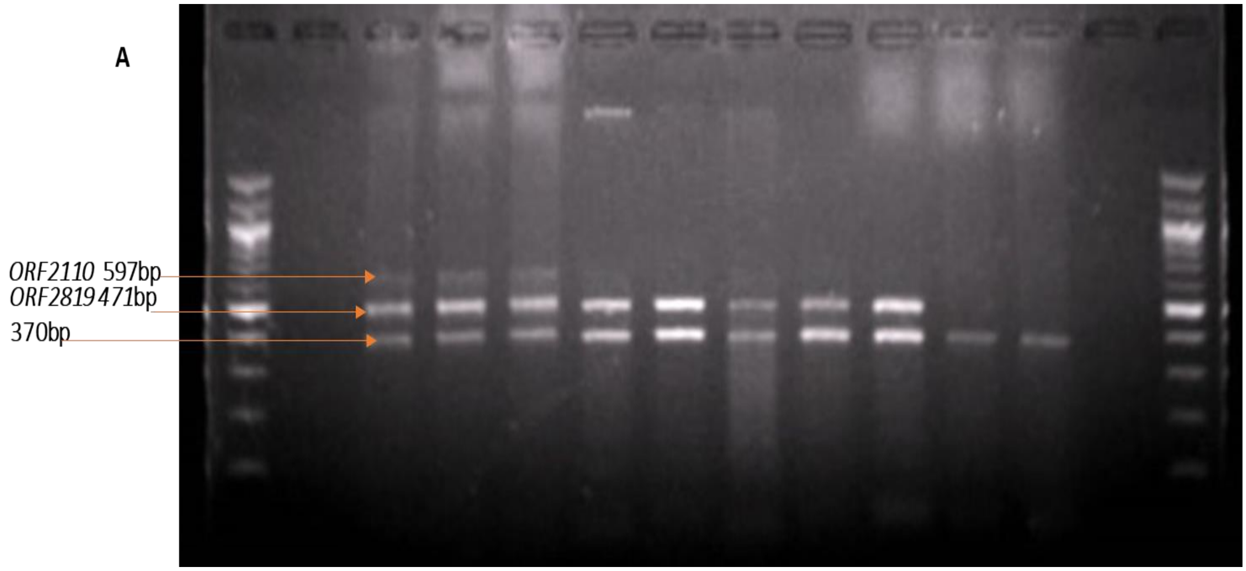

raw gel for the amplification of *L. monocytogenes* serotypes

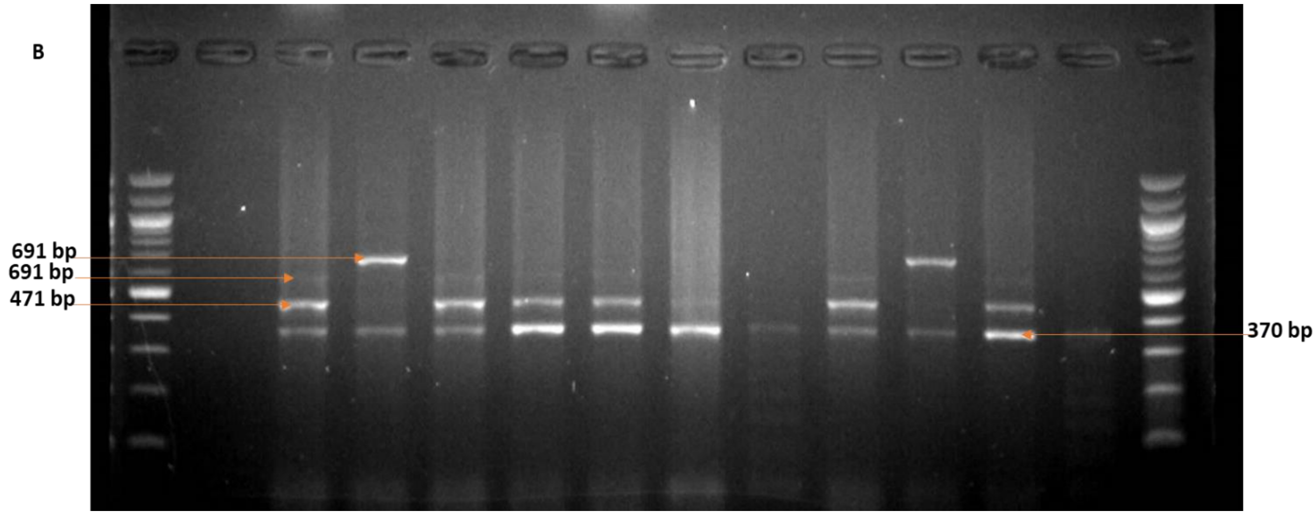

raw gel for the amplification of *L. monocytogenes* serotypes

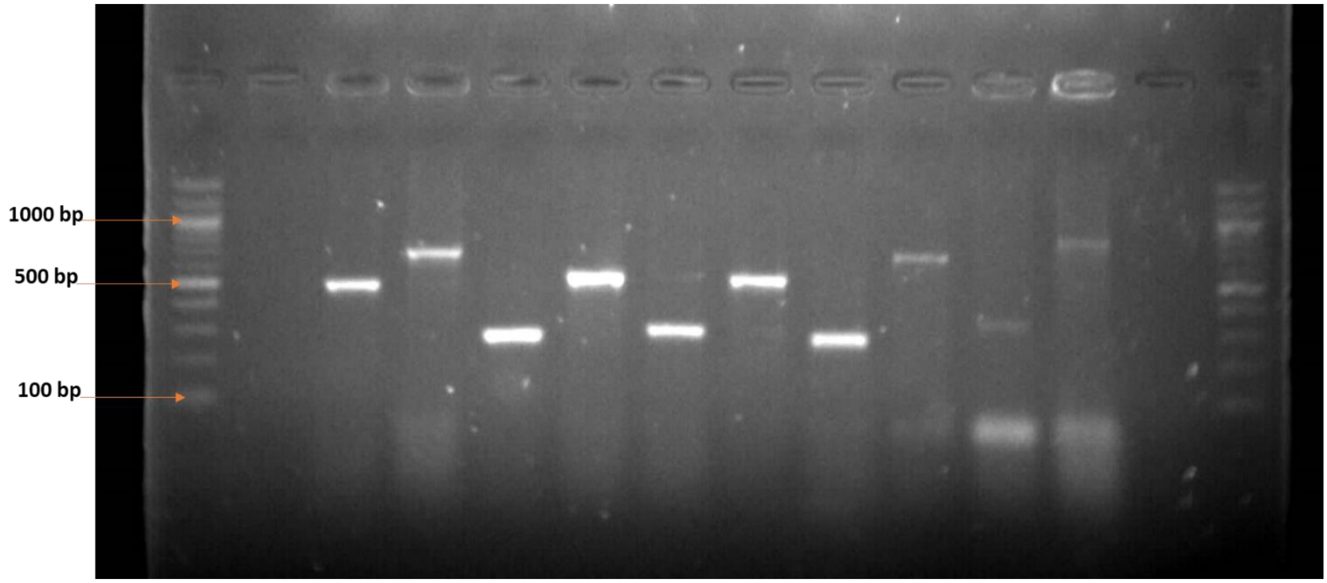

raw gel for the amplification of *L. monocytogenes* virulence genes
