# Supplementary material for: Assessment of the molecular epidemiology and genetic multiplicity of Listeria monocytogenes recovered from ready-to-eat foods following the South African listeriosis outbreak
Source: Sci Rep. 2022 Nov 22;12:20129. doi: 10.1038/s41598-022-20175-x (PMC9684121; doi:10.1038/s41598-022-20175-x)
Supplement: Supplementary file 2 — Supplementary Information 2. [file 41598_2022_20175_MOESM2_ESM.pdf]

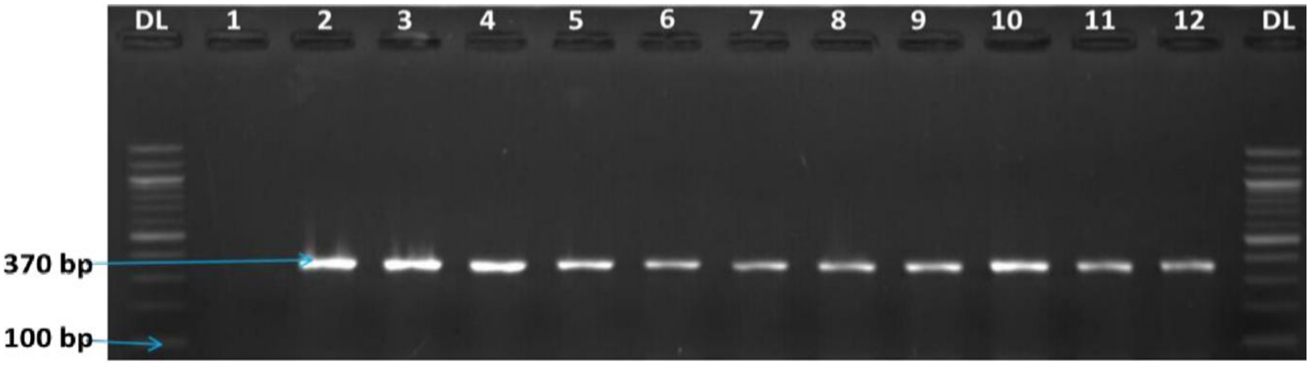

Figure S1a. Agarose gel electrophoresis of the DNA fragment generated by the simplex PCR to identify the *prs* gene (370 bp) of the *Listeria* genus. Lane DL: 100 bp DNA ladder, lane 1: -ve control, lane 2: +ve control (*L. monocytogenes* ATCC 19118), lane 3-12 positive *Listeria* genus.

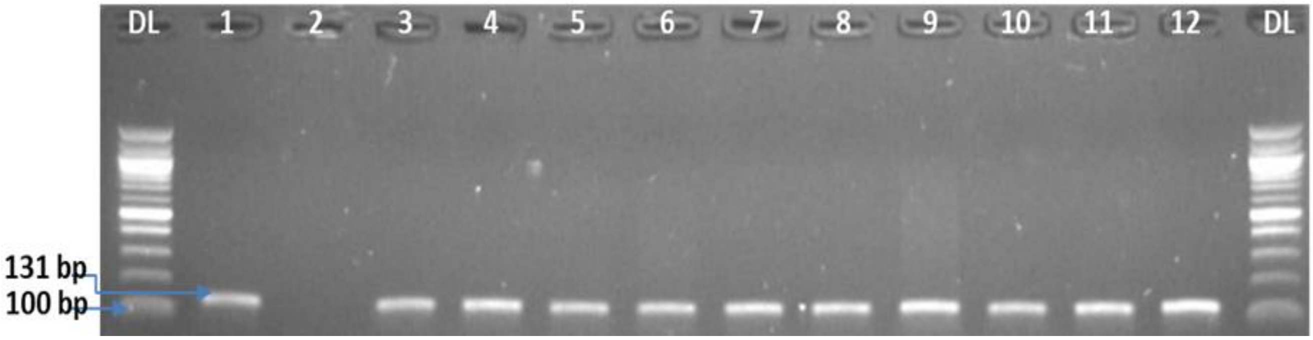

Figure S1b. Agarose gel electrophoresis of the DNA fragment generated by the simplex PCR for the amplification of the *iap* gene fragment (131 bp) for the confirmation of *L. monocytogenes*. Lane DL: 100 bp DNA ladder, lane 1: +ve control, lane 2: -ve control (*L. monocytogenes* ATCC 19118), lane 3-12 positive *L. monocytogenes* isolates.

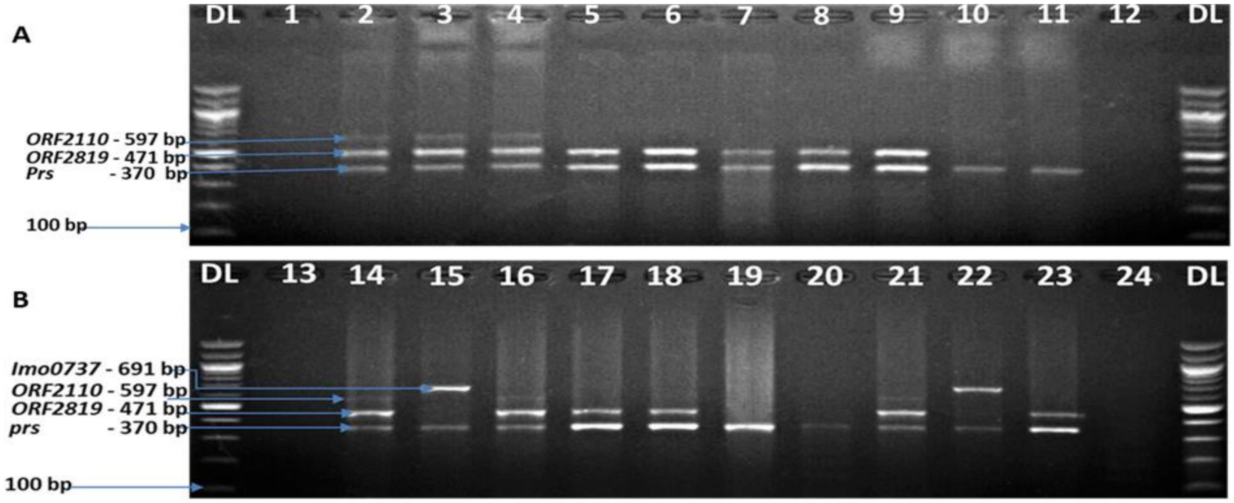

Figure S2. Agarose gel electrophoresis of the DNA fragments generated by the multiplex PCR of positive serotypes 1/2a, 1/2b and 4b strains of *L. monocytogenes.* Lane DL:100 bp DNA ladder; lane 1, 12, 13 and 24: -ve controls; lane 2 – 4, 14, 16 and 21: positive serotype 4b strains; lane 5 – 9, 17, 18 and 23: positive serotype 1/2b strains, lane 15 and 22: positive serotype 1/2a strains. All *L. monocytogenes* strains amplified the *prs* gene fragment. Genes corresponding to the amplified fragment and their molecular sizes are shown on the left.

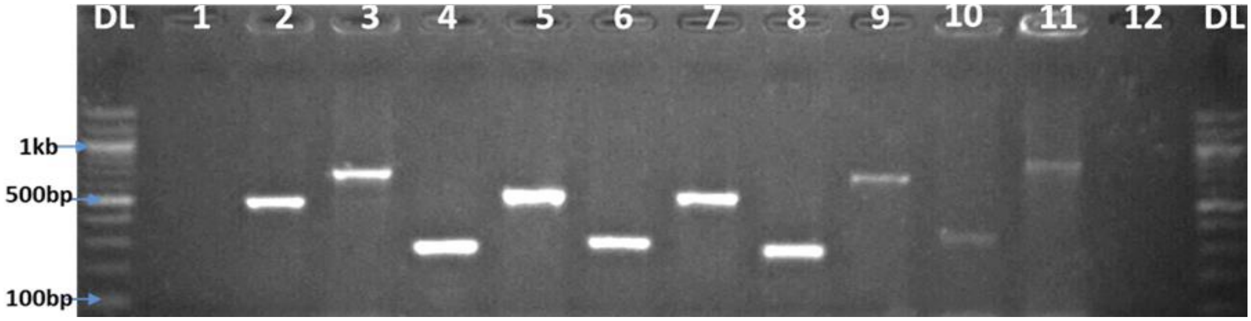

Figure S3. Agarose gel electrophoresis of the amplified DNA fragment of LIPI-1 and LIPI-2 virulence genes of *L. monocytogenes*. Lane M: 100 bp DNA ladder, lane 1 and 12: -ve control, lane 2: *hly* (496 bp), lane 3: *plc*A (674 bp), lane 4: *inl*A (256 bp), lane 5: *inl*C (517 bp), lane 6: *inl*B (272 bp) lane 7: *prf*A (479 bp), lane 8: *inlI*J (238 bp), *act*A (650 bp), *plc*B (320 bp), and *mpl* (798 bp).

Table S1. Primer sets for the amplification of *Listeria monocytogenes* of virulence genes

Targeted genes	Primer	Sequence (5′-3′)	Product size (bp)	Reference
prs	*Prs*-F	GCTGAAGAGATTGCGAAAGAAG	370	(Doumith et al., 2004)
	Prs-R	CAAAGAAACCTTGGATTTGCGG		
iap	*iap*-F	ACAAGCTGCACCTGTTGCAG	131	Furrer et al., 1991
	iap-R	TGACAGCGTGTGTAGTAGCA		
*inl*A	*inl*A-F	CCTAGCAGGTCTAACCGCAC	256	(Coroneo et al., 2016)
	*inl*A-R	TCGCTAATTTGGTTATGCCC		
*inl*B	*inl*B-F	TGATGTTGATGGAACGGTAAT	272	(Du et al., 2017)
	*inl*B-R	CTCGTGGAAGTTTGTAGATGC		
*inl*C	*inl*C-F	AATTCCCACAGGACACAACC	517	(Liu et al., 2007)
	*inl*C-F	CGGGAATGCAATTTTTCACTA		
*inl*J	*inl*J-F	TGTAACCCCGCTTACACAGTT	238	(Liu et al., 2007)
	*inl*J-R	AGCGGCTTGGCAGTCTAATA		
*prf*A	*prf*A-F	AACCAATGGGATCCACAAG	479	(Jung et al., 2009)
	*prf*A-R	ATTCTGCTAACAGCTGAGC		
hly	*hly*-F	CAAACTGAAGCAAAGGATGCA	496	(Jung et al., 2009)
	hly-R	CTAATGTATTTACTGCGTTGTTA		
*plc*A	*plc*A-F	CAGCATACTGACGAGGTGTG	674	(Jung et al., 2009)
	*plc*A-R	GATGTCCGCTCTACCTGA		
*plc*B	*plc*B-F	GCATGATATTGACAGCAAATTA	320	(Jung et al., 2009)
	*plc*B-R	TGAAATACTTTGCTCCTGTT		
mpl	*mpl*-F	TGTATCATCATGGTAATAGCT	798	(Jung et al., 2009)
	mpl-R	TGGATCCGTAAACATATTCGT		
*act*A	*act*A-F	CCAAGCGAGGTAAATACGGGA	650	(Lomonaco et al., 2012)
	*act*A-R	GTCCGAAGCATTTACCTCTTC		
